# Expression of innate immune complement regulators on brain epithelial cells during human bacterial meningitis

**DOI:** 10.1186/1742-2094-3-22

**Published:** 2006-09-02

**Authors:** Cecile Canova, Jim W Neal, Philippe Gasque

**Affiliations:** 1 Brain Inflammation and Immunity Group, Department of Medical Biochemistry, Cardiff University, Heath Park, Cardiff, CF14 4XN, UK; 2 Department of Pathology, Neuropathology Laboratory; Cardiff University, Heath Park, Cardiff, CF14 4XN, UK; 3 LBGM, Faculty of Sciences and Technologies, University of la Reunion, 15 Avenue René Cassin, BP7151, 97715, Saint Denis, Reunion

## Abstract

**Background:**

In meningitis, the cerebrospinal fluid contains high levels of innate immune molecules (e.g. complement) which are essential to ward off the infectious challenge and to promote the infiltration of phagocytes (neutrophils, monocytes). However, epithelial cells of either the ependymal layer, one of the established niche for adult neural stem cells, or of the choroid plexus may be extremely vulnerable to bystander attack by cytotoxic and cytolytic complement components.

**Methods:**

In this study, we assessed the capacity of brain epithelial cells to express membrane-bound complement regulators (ie, CD35, CD46, CD55 and CD59) *in vitro *and in situ by immunostaining of control and meningitis human brain tissue sections.

**Results:**

Double immunofluorescence experiments for ependymal cell markers (GFAP, S100, ZO-1, E-cadherin) and complement regulators indicated that the human ependymal cell line model was strongly positive for CD55, CD59 compared to weak stainings for CD46 and CD35. In tissues, we found that CD55 was weakly expressed in control choroid plexus and ependyma but was abundantly expressed in meningitis. Anti-CD59 stained both epithelia in apical location while increased CD59 staining was solely demonstrated in inflamed choroid plexus. CD46 and CD35 were not detected in control tissue sections. Conversely, in meningitis, the ependyma, subependyma and choroid plexus epithelia were strongly stained for CD46 and CD35.

**Conclusion:**

This study delineates for the first time the capacity of brain ependymal and epithelial cells to respond to and possibly sustain the innate complement-mediated inflammatory insult.

## Background

The activation of complement is an important component of the innate immune response providing the capacity to detect and to clear pathogens (for review [1]). The main source of complement proteins is the liver, but many cell types including fibroblasts, epithelial and endothelial cells as well as glia and neurons also synthesise most of the complement components [2]. The activation of one of three different complement pathways, the classical, alternative or mannan binding lectin pathways, leads to the formation of C3 and C5 convertases [1]. Some of the resulting compounds, called opsonins, bind to pathogens allowing the formation of the membranolytic attack complex (MAC) [3,4]. During the acute phase of inflammation, complement fragments may also bind to the surface of resident host cells and promote bystander effects with the formation of cytotoxic and cytolytic MAC. Under these conditions, many cell types facing complement attack express several key complement regulators (CRegs) on their membranes to avoid damages by inhibiting either the C3 convertases (complement receptor type 1, CR1/CD35; membrane cofactor protein, MCP/CD46; decay accelerating factor, DAF/CD55) or by avoiding the formation of MAC (CD59). In the human brain, astrocytes, microglia and oligodendrocytes express CRegs (for review [5,6]) but neurons are extremely susceptible to complement mediated lysis as they express low levels of CRegs [7,8].

The expression of CRegs by the different brain epithelial cells remains poorly characterized. Very high levels of complement proteins are present in the cerebrospinal fluid (CSF) particularly in infection or inflammatory conditions of the brain [9] and presumably as a consequence of plasma transudation or intrathecal synthesis by infiltrating leukocytes and resident activated epithelial cells [10,11]. The epithelium lining brain ventricles (ependyma), spinal cord and choroid plexus consists of specialized glial cells (for review [12]) and ependymal cells of the ventricles express the glial fibrillary acidic protein (GFAP) and S100 markers [13]. In adult, neural stem cells reside within the ependyma and/or subependyma (also known as the subventricular zone, SVZ) and, from these proliferative zones, cells migrate to their destiny in the injured brain where they differentiate into neurons and glia [13-15]. Remarkably, ependymal cells contribute to a unique and heterogeneous epithelial layer providing a critical physical barrier against pathogen infiltration and cell-mediated cytotoxicity (e.g. neutrophil compounds). In bacterial meningitis, the levels of complement anaphylatoxins C3a and C5a are dramatically increased in the CSF enhancing the influx of inflammatory cells into the ventricle and increasing complement biosynthesis and promoting activation [11,16,17]. Critically, the epithelial cells exposed to the CSF have to withstand the activities of strong and sustained complement activation.

Histopathological assessment together with electron microscopy studies (this study) have revealed that the ependymal cells in meningitis appear resistant to the potential toxic effects of microbial and neutrophil products. In contrast, it has been reported that in severe bacterial and fungal ependymitis, the layer of epithelial cells is highly destructed [18]. The ependyma is vulnerable to injury throughout both fetal and adult life and particularly in diseased conditions but the cellular and molecular nature of the intrinsic mechanisms conferring resistance (or not) to tissue damage remains poorly characterised.

To explore the capacity of brain epithelial cells (i.e. choroid plexus as well as ependymal layer lining the ventricle) to protect themselves from severe complement attack in disease conditions we have investigated and compared the expression of CRegs between control and several meningitis cases. Moreover, a human ependymoma primary culture model was established to provide additional information about Cregs expression by ependymal cells in culture.

## Methods

### Source of tissues

**Figure 1:**
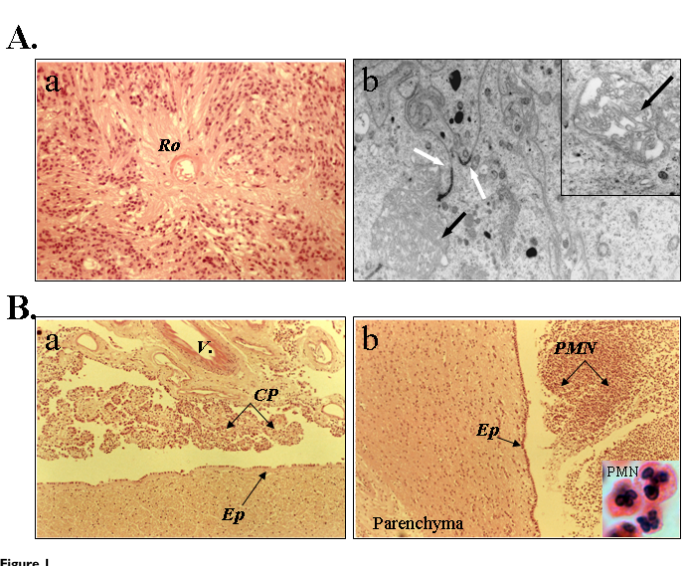
Structural and ultrastructural (electron microscopy) analyses of human brain tissue sections obtained from the ependymoma (case 9945) and meningitis cases. Aa. H&E staining of paraffin embedded wax tissue sections; original magnification, ×200. The presence of perivascular rosette (Ro) formation is typical of an ependymoma. Ab. Electron micrographs taken of the araldite-enhanced ependymoma cells. Original magnification, ×13500; inset: original magnification, × 23000. The white arrows indicate junction complexes between cells and the black arrows indicate microvilli (see inset). These structures reveal key characteristics of ependymal cells. Ba, Bb, Meningitis cases. Original magnification, ×100. Choroid plexus (a) and ependyma (Ep) (b) show a continuous layer of intact epithelial cells despite the presence of neutrophils (PMN) inside the ventricle (particularly in panel Bb, see inset, magnification ×1000). V, vessel; Cp, choroid plexus; Ep, ependymal layer.

Small blocks of paraffin wax embedded temporal lobe, containing the hippocampus and the choroid plexus from the lateral ventricles were available for examination from cases of meningococcal meningitis. From the same cases, small tissue blocks from the caudate nucleus, lined by ependymal cells, were also available. In all cases, there was significant numbers of neutrophils within the cerebrospinal fluid in contact with the lining ependyma and within the ventricle system (see Figure 1Bb). Blocks of hippocampus with choroid plexus, together with blocks of caudate nucleus lined by ependymal cells were available from control cases without evidence of systemic infection, cerebral ischemia or neurodegeneration. Control cases did not present astrogliosis and microgliosis as indicated by the GFAP and HLA-II stainings, respectively. Tangles and βA4-plaques were not present in control cases. All cases were available from the Neuropathology laboratory (JWN, Cardiff University, Heath Hospital, Cardiff) and used under the guidelines approved by the Bro Taf Health Authority local ethical approval (reference 98/2773).

Tissues from each case had been fixed in 2% neutral buffered formalin for two weeks, subsequently processed in paraffin wax and sections 6 μm-thick cut and stained for further light microscopy and immunocytochemical investigations.

### Primary cell cultures of ependymoma cell line (clone 9945)

A primary culture of ependymal cells was prepared from a fragment of a biopsy from a spinal cord ependymoma.

Briefly, tissue was minced in MEM-medium with iridectomy scissors. Subsequent trypsinization (0.025% trypsin in calcium and magnesium free phosphate-buffered saline) was performed for 15 min at 37°C. After removing trypsin by centrifugation the cells were resuspended in RPMI medium (Gibco) supplemented with 10% foetal calf serum/L-glutamine 1X/streptomycin (100 μg/ml)/penicillin (62.5 μg/ml) and dissociated by mechanical trituration using a Pasteur pipette in order to obtain a suspension of single cells. Cells were plated and cultured at 37°C in a humidified 5% CO_2 _incubator (Heraeus, Hanau, Germany).

### Electron microscopy of ependymoma cells

A small fragment of tissue from the ependymoma sample was dissected and placed in a solution of 2.5% glutamine/3% osmium/Araldite resin. Further processing for ultrastructural electron microscopy was carried out as previously described [19].

### Source of antibodies

Mouse anti-HLA Class II (clone CR3/43, M0775), rabbit anti-GFAP (Z0334) and the FITC- and Rhodamine-coupled secondary antibodies, goat anti-mouse IgG and goat anti-rabbit IgG, were obtained from DAKO Ltd. (High Wycombe, Bucks, United Kingdom). Rabbit antibodies against CD59, CD35, CD55, and CD46 were all raised in house after immunization using purified human CRegs [20]. Mouse monoclonal anti-CD59 (clone BRIC 229) and anti-CD55 (clone BRIC 216) were from the International Blood Group Reference Laboratory (IBGRL, Elstree, Herts, UK). Mouse anti-CD35 was from Dako and mouse anti-CD46 was from Serotec (Oxford, UK) The peroxidase-conjugated secondary antibodies goat anti-mouse IgG and goat anti-rabbit IgG were from Bio-Rad (Hermel Hempstead, Hertfordshire, United Kingdom).

### Immunohistochemistry

6 μm thick tissue sections were mounted on super-frost glass slides (Surgipath Europe Ltd., Peterborough, United Kingdom). Antigen retrieval was required for all anti-CRegs antibody staining protocols. To this aim, sections were heated in freshly prepared 0.2% citric acid buffer at pH 6.0 for 30 min in a microwave at full power (750 watts). Sections were left 30 min at room temperature and then rinsed in tap water.

Sections were immunostained by the indirect immunoperoxidase/3'3' diaminobenzidine HCl method. All sections were incubated overnight with their appropriate antibodies diluted in 1% BSA prepared in phosphate buffer. The secondary antibodies were similarly prepared and used at 1:200 (1 to 2.5 ug/ml final). Sections were washed 3 times in PBS 1× after which they were developed for 5 minutes in a freshly made solution of 0.05% diaminobenzidine (DAB) and 0.005% (v/v) hydrogen peroxide diluted in PBS 1×. After a wash in tap water, sections were counterstained using hematoxylin. After a full dehydration in ethanol, the sections were cleared in xylene and mounted.

### Immunocytochemistry

Ependymoma cells were cultured in RPMI medium supplemented with 10% foetal calf serum on poly-D-lysine-coated coverslips for 2 days. Then, after 5 washes in NaCl 0.9%, cells were fixed in cold acetone. The phenotype of the cells was further assessed by staining the coverslips with polyclonal antibody against GFAP (Dako Ltd, High Wycombe, Bucks, United Kingdom) and monoclonal antibodies against S-100 protein (Sigma, Saint Louis, Missouri, USA) ZO-1 (zonula occludens 1) and E-cadherin (Becton Dickinson, Oxford UK) [12,21]. The coverslips were also stained with primary rabbit and mouse antibodies against human CRegs 1 h at room temperature. After three washes in PBS 1×, they were incubated for 1 h at room temperature with 4'-6-diamino-2-puenylindole-2 HCl (DAPI; 1:1000, nuclear staining) and FITC-coupled goat anti-mouse IgG or rhodamine-coupled goat anti-rabbit IgG (1:100). Coverslips were washed 3 times in PBS 1× and then mounted with Vectashield medium (Vector laboratories, Peterborough, UK) on a glass slide.

## Results

### Histopathological assessment of the human ependymoma (case 9945) and meningitis cases

All clinical samples used in the study were first thoroughly analysed for histopathology hallmarks. Histological features of the ependymoma revealed the presence of perivascular rosette formation with acellular areas (Figure 1Aa, *Ro*) and peripheral individual cells with vesicular nuclei. There was no evidence of either necrosis, mitosis or endothelial proliferation. On this basis, the tumour was classified as WHO grade II [22]. Further immunohistological examinations of paraffin embedded tissue sections indicated that the ependymoma was strongly stained for GFAP and S100 (data not shown). Electron micrographs were taken of the Araldite-enhanced specimen on a Joel Electron micrograph, at 27000 and 13500 magnifications (Figure 1Ab). The ultrastructural features were of a tumour with junctural complexes between adjacent cells (white arrows). A small rosette-like structure containing cells with apical microvilli was also present (black arrow, inset). The overall ultrastructural findings were characteristic of an ependymoma [23]. These tumors are extremely rare and we were able to establish primary cultures of ependymal cells isolated from the same biopsy used for histology (see below).

We also performed histopathological assessments of the meningitis cases. Figure 1Ba–b depicts the level of polymorphonuclear (PMN) infiltration within the brain ventricles closed to remarkably well preserved epithelia of the choroid plexus and the ependymal layer. Although control brains were free of any infiltrating leukocytes, we found robust PMN infiltration in all bacterial meningitis cases. The local inflammation was associated with a strong GFAP staining of ependymal cells (Table 1). Interestingly, Kolmer cells (macrophage-like cells) but not the epithelial cells were found to express high levels of HLA-class II (Table 1).

**Table 1:** History, pathology and immunostaining data of control and meningitis cases for complement regulatory proteins and cell markers.

***Gender***	***Age (yr)***	***PM interval (hours)***	***Cases***	**Immunodetection of complement regulatory proteins**								Inflammatory index		
				
				**CD55**		**CD59**		**CD46**		**CD35**		**HLA-II**		**GFAP**
				
				**CP**	**Ep**	**CP**	**Ep**	**CP**	**Ep**	**CP**	**Ep**	**CP**	**Ep**	**Ep**
M	52	48	normal	+	+	+	+	+	0	0	0	++ (K)	0	+
M	86	48	normal	+	+	+	+	0	0	0	0	+ (K)	0	+
M	23	12	bacterial meningitis	++	+	++	+	++	0	+	+	++ (K)	0	+++
F	68	48	bacterial meningitis	+	++	++	++	++	++	++	+	++ (K)	0	+++
F	55	48	bacterial meningitis	+++	na	++	na	+++	na	++	na	++ (K)	0	na

Abbreviations used: CP, choroid plexus epithelial cells; Ep, ependymal cell layer; M, male; F, female; K, Kolmer cells; 0: negative; +: very few cells positive; ++: positive; +++: strongly positive. (PM, Postmortem); na: not applicable

### Tumour-derived ependymal cells (clone 9945) express several key complement regulators

**Figure 2:**
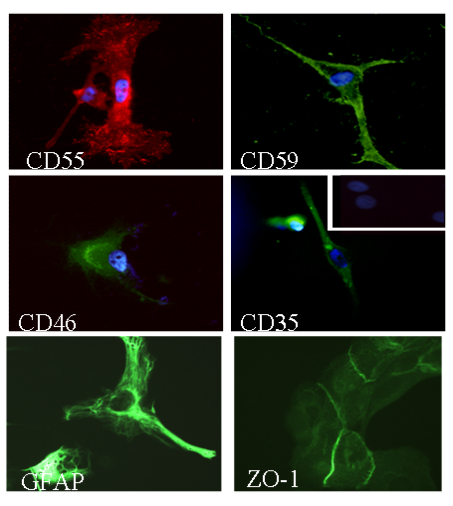
Immunofluorescence analyses of the human tumour-derived ependymoma primary cultures (clone 9945) stained for complement regulatory proteins and cell markers. Cells on coverslips were fixed with acetone and stained with antibodies against ependymal cells specific markers (GFAP, ZO-1 and S100, not shown) and complement regulators proteins (CD55, CD59, CD46 and CD35). Original magnification ×400. Background staining was observed using irrelevant antibodies (inset). Nuclei were counterstained with DAPI (blue).

Ependymal primary cultures were analysed for specific cell markers and CRegs by double immunofluorescence staining experiments. The large majority of cells presented a typical ependymal cell phenotype and were strongly stained for GFAP and S100 (data not shown). CD11b+, CD14+ contaminating Kolmer cells were not identified in our cultures. However, epithelial-like cells demonstrated strong membrane staining with the anti-CD55 and CD59. In contrast, they were weakly stained for CD46 and CD35 (Figure 2). These data were confirmed using different cell culture passages (1–5). FACS analyses could not be performed given the limited number of cells isolated from the ependymoma.

### The expression of several key regulators of the complement system is dramatically upregulated in choroid plexus epithelium in meningitis

**Figure 3:**
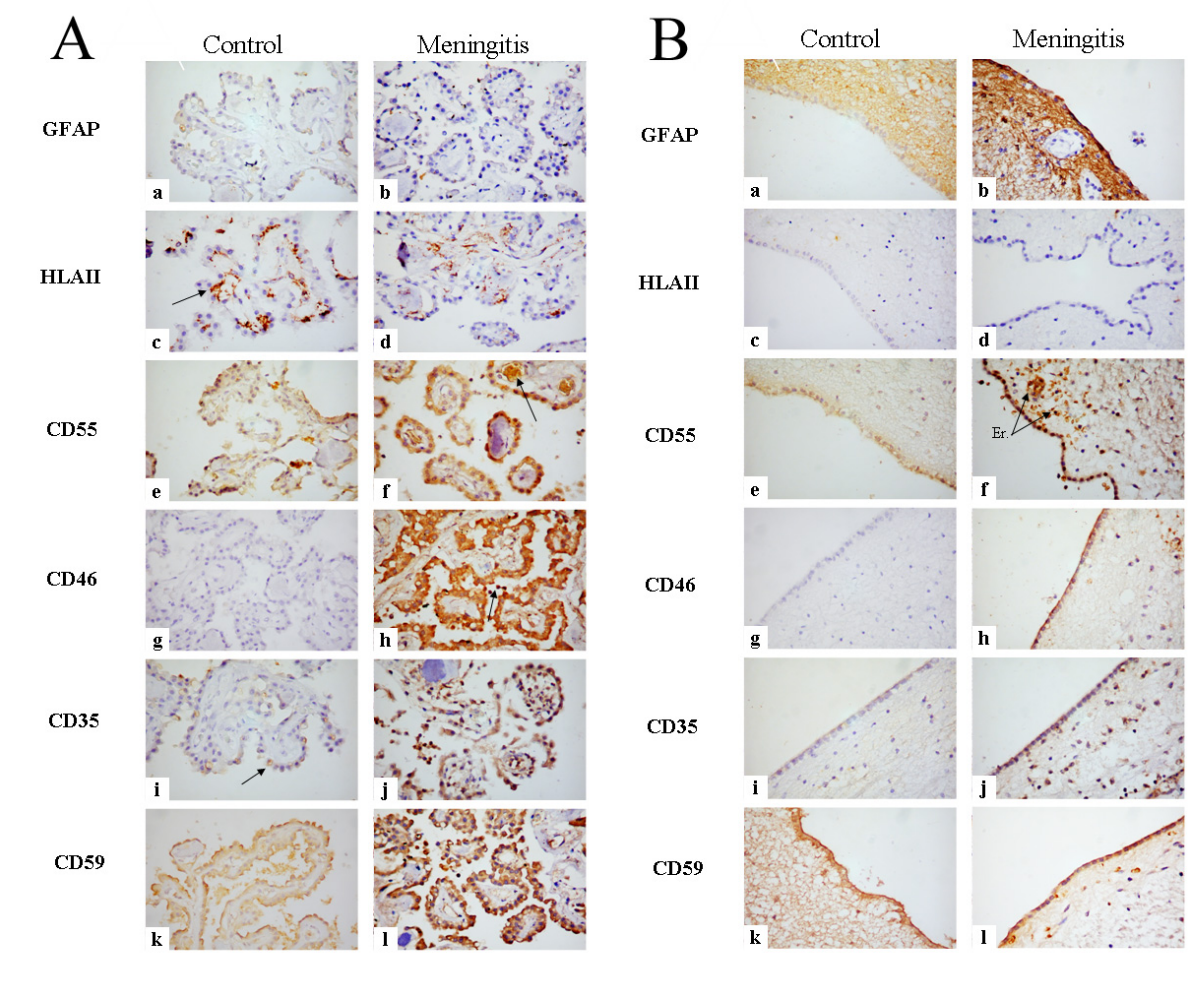
Immunoperoxidase histochemistry analyses of paraffin-wax sections to assess the expression of complement regulatory proteins (CRegs) in control and meningitis cases. Rehydrated paraffin wax sections of human choroid plexus in normal and meningitis cases immunostained with antisera to inflammatory cells and to membrane regulators proteins. Original magnification, ×400. Panel A: Choroid plexus staining data: a and b, Rabbit anti-GFAP. c and d, Rabbit anti-HLA II staining only Kolmer cells (arrow). No differences are noticed between normal and meningitis cases. e and f, Rabbit anti-CD55. Choroid plexus epithelium in normal cases is weakly stained (e) but more strongly in meningitis cases (f). Erythrocytes in f are stained for CD55 (arrow). g and h, Rabbit anti-CD46. Epithelium is weakly stained in normal cases (g) but strongly in meningitis epithelia, while infiltrating PMN (arrow) are strongly CD46+ (h). i and j, Rabbit anti-CD35. Weak staining is detected on Kolmer cells in normal sections (i) but a strong staining is noticed in meningitis epithelia and infiltrating PMNs (j). k and l, Rabbit anti-CD59. Normal choroid plexus epithelia are stained for CD59 (k) and with a stronger staining in meningitis (l). Panel B: same as above but assessing the expression of CRegs on ependymal cells. Note that erythrocytes (Er) within blood vessels are strongly stained for CD55 (f).

We next analysed the capacity of epndymal cells and the epithelial cells of the choroid plexus to express Cregs in healthy and inflamed brains. Of important note, we observed that the epithelium of the choroid plexus and ependymal cells lining the ventricles in meningitis was largely preserved despite the presence of large number of neutrophils in the ventricles (Figure 1B and Figure 3). Control and meningitis cases were immunostained for all membrane-bound CRegs and the level of staining was scored by three independent examiners blinded to the individual treatment groups (Table 1 and Figure 3A). Kolmer cells in choroid plexus stroma were strongly stained using an antibody to HLA Class II (clone CR3/43) but no differences in the intensity or pattern of staining were noticed between control and meningitis cases (Figure 3A, a/b).

Epithelial cells of the choroid plexus were clearly negative for GFAP. Affinity purified polyclonal antibodies against CRegs were used on paraffin-embedded tissue sections. First, the staining with anti-CD55 antibody was weak in the choroid plexus in control cases but was more prominent in one case of meningitis (Figure 3Ae/f,; Table 1). Anti-CD46 staining showed a significant increase between normals compared to all meningitis case (Figure 3Ag/h; Table 1). Interestingly, CD35 was solely expressed by Kolmer cells in normal choroid plexus epithelium (Fig. 3Ai). In contrast, during meningitis, ependymal and Kolmer cells were strongly stained for CD35 (Figure 3Ai/j; Table 1). The apical CD59 staining was much pronounced on epithelial cells of the choroid plexus in pathological conditions (Figure 3Ak/l; Table 1).

### Overexpression of CD46 and CD35 in ependymal cells from meningitis cases

Ependymal cells from ventricle lining showed staining differences compared to the choroid plexus from the same cases. Some anti-GFAP staining was detected in normal conditions but the immunostaining was highly increased in meningitis cases (Figure 3B, Table 1). Ependymal cells did not show any HLA Class II staining either in normal or pathological cases (Figure 3B, Table 1). Ependymal cells express CD55, CD59 and CD46 antigens in normal cases and the stainings were increased in meningitis cases (Figure 3B; Table 1). Interestingly CD35 expression was not detected on ependymal cells of control brains but was expressed in meningitis cases (Figure 3B, Table 1).

## Discussion

The organisation of brain epithelia is very similar to most other epithelial membranes as they form a cellular network tightly interconnected by gap junctions [24]. Despite the presence of these physical barriers, it is now well established that several micro-organisms can infiltrate the meninges and choroid plexus and gain access to the brain parenchyma [25]. Moreover, these infectious challenges will promote a sustained cellular and molecular innate immune responses, the local production of several key cytotoxic and cytolytic proteins (complement, TNFα, defensins) and with the potential to harm the surrounding neural cells [26-30]. Several elegant studies have demonstrated a pro-inflammatory reaction of ependyma and choroid plexus epithelia in response to bacterial infection including the expression of tumour necrosis factor-α [31,32] and ICAM-1 to facilitate neutrophil invasion [22,33]. Whether the ependyma and choroid plexus are able to control these inflammatory insults may be important to the plasticity and homeostasis of the inflamed brain. Remarkably, comprehensive structural and ultrastuctural analyses of meningitis cases (illustrated in Figure 1Bb and JWN's unpublished data) indicated that the epithelial cells remained largely unaffected. This study was undertaken to decipher some of the intrinsic pathways expressed by reactive brain epithelial cells to control bystander cytotoxic properties of the local innate immune response.

Our data indicate that the integrity of the ependymal and choroid plexus layers was preserved despite the large number of neutrophils in the CSF while the expression of CRegs on both epithelia was dramatically increased during meningitis. We found that the level of all membrane-bound regulators was dramatically upregulated on choroid plexus epithelial cells and ependyma in all meningitis cases. The regulation of CD55 expression by epithelial cells of the choroid plexus demonstrated minor changes between control and meningitis cases. In contrast, levels of CD55 and CD46 were strongly elevated on ependymal cells in disease conditions. These data argue for a regional specificity and independent regulation of Cregs between epithelial cells of the choroid plexus and the ependyma of the ventricle which may be due to the local inflamed microenvironment.

The mechanisms controlling the expression of Cregs on brain epithelial cells are largely ill-characterised. The expression of Cregs has been studied on several cell types including human vascular endothelial cells and was shown to be regulated by a plethora of proinflammatory cytokines (e.g. TNF, IL1) and LPS [34]. In meningitis, both epithelia are exposed to inflammatory cytokines (TNF-α), to complement-derived products (e.g. C3a, C5a and sublytic doses of C5b9) as well as bacterial products such as lipopolysaccharide (LPS) and peptidoglycans (PGs). Together these compounds may profoundly affect the plasticity of the brain epithelial cells and potentially driving robust expression of regulatory proteins to protect from bystander complement attack. In human meningitis, we found that epithelial cells and glial cells of the subependyma failed to express MHC class II antigens but in contrast, were strongly stained for GFAP, a classical marker of ependymal cell activation. Interestingly, the expression of TLR4 mRNA in choroid plexus epithelium has been reported in normal rat brain [35] and correlated with CD14 mRNA expression [36]. Our preliminary unpublished observations confirmed that human brain epithelial cells of the choroid plexus are strongly stained for TLR4 and CD14 while ependymal cells were solely CD14+. The presence of these two key pattern recognition receptors of the innate immune system raises the possibility that brain epithelial cells are capable of sensing carbohydrate structures released from bacterial cell wall (LPS, PGs) [37,38]. It remains to be ascertained whether the treatment of brain epithelial cells with LPS and/or PGs can control the level of Cregs expression and experiments along these lines are now highly warranted.

It is important to emphasise that a delicate balance probably exits between the anti-inflammatory/protective responses to protect the brain against the rather pro-inflammatory/toxic response in severe bacterial meningitis. The severity of the lesioned microenvironment may determine ependymal cell survival and ultimately, the clinical outcome with associated sequelae. The final response will involve the proliferation and differentiation of the neural stem cells which again could be affected by inflammatory mediators.

New data emphasise the key role of brain epithelial cells to integrate and further orchestrate the local innate immune response with the production of innate components of the complement system while preventing secondary tissue damage. Of note, the increased expression of CRegs by brain epithelial cells may also contribute to a double-edged sword scenario. On the one hand, high levels of membrane-bound and soluble Cregs from brain epithelial cells would certainly confer increased protection from complement-mediated attack but on the other, bacteria and viruses (e.g Measles) are known to bind to several Cregs and so evading the host innate immune defense mechanisms [29,39-41].

A better understanding of the cellular and molecular innate immune responses in the CNS and deciphering the pathways involved in the cross-talk between brain epithelial cells and infectious agents will help enormously to develop novel therapeutic strategies against brain infection [42].

## Conclusion

Our findings underscore the remarkable capacity of brain epithelial cells to withstand complement activation and to survive within an inflammatory site. The Cregs on brain epithelial cells may on one hand help to protect from bystander complement attack but on the other provide a niche for bacterial infection and contributing to meningitis pathology.

## Abbreviations

MAC: Membrane attack complex

CRegs: Complement regulators

CSF: Cerebro spinal fluid

GFAP: Glial fibrillary acidic protein

CR: complement receptor

MCP: membrane cofactor protein

DAF: Decay accelerating factor

## Competing interests

The author(s) declare that they have no competing interests.

## Authors' contributions

Dr Canova was involved with the day-to-day experimental approach and the analyses of the data. Histopathological assessment was performed by Dr Jim Neal, Neuropathologist at Cardiff University, Medical School. Prof. Philippe Gasque was involved with the design and the supervision of the work; preparation of the manuscript was done by Dr Canova/Prof. Gasque.
